# Identifying conformational changes with site-directed spin labeling reveals that the GTPase domain of HydF is a molecular switch

**DOI:** 10.1038/s41598-017-01886-y

**Published:** 2017-05-10

**Authors:** Laura Galazzo, Lorenzo Maso, Edith De Rosa, Marco Bortolus, Davide Doni, Laura Acquasaliente, Vincenzo De Filippis, Paola Costantini, Donatella Carbonera

**Affiliations:** 1 Department of Biology, University of Padova, Viale G. Colombo 3, 35131 Padova, Italy; 2 Department of Chemical Sciences, University of Padova, Via F. Marzolo 1, 35131 Padova, Italy; 3 Department of Pharmaceutical and Pharmacological Sciences, University of Padova, Via F. Marzolo 5, 35131 Padova, Italy

## Abstract

[FeFe]-hydrogenases catalyse the reduction of protons to hydrogen at a complex 2Fe[4Fe4S] center called H-cluster. The assembly of this active site is a multistep process involving three proteins, HydE, HydF and HydG. According to the current models, HydF has the key double role of scaffold, upon which the final H-cluster precursor is assembled, and carrier to transfer it to the target hydrogenase. The X-ray structure of HydF indicates that the protein is a homodimer with both monomers carrying two functional domains: a C-terminal FeS cluster-binding domain, where the precursor is assembled, and a N-terminal GTPase domain, whose exact contribution to cluster biogenesis and hydrogenase activation is still elusive. We previously obtained several hints suggesting that the binding of GTP to HydF could be involved in the interactions of this scaffold protein with the other maturases and with the hydrogenase itself. In this work, by means of site directed spin labeling coupled to EPR/PELDOR spectroscopy, we explored the conformational changes induced in a recombinant HydF protein by GTP binding, and provide the first clue that the HydF GTPase domain could be involved in the H-cluster assembly working as a molecular switch similarly to other known small GTPases.

## Introduction

Biohydrogen production, one of the most promising frontiers in the field of renewable energies, is achieved in nature by several prokaryotic and eukaryotic microorganisms through a general class of evolutionarily unrelated metalloenzymes called hydrogenases. Among them, [FeFe]- and [NiFe]-hydrogenases are the most widespread^1^. These proteins have different distribution, catalytic properties and molecular architectures, but they are both able to reversibly reduce protons to H_2_ by means of metal clusters with some key similarities, including the coordination to the polypeptide chain through four conserved cysteine residues and the presence of iron-bound CO and CN^−^ ligands^2^. In the case of [FeFe]-hydrogenases, the active site (referred to as the H-cluster) is particularly complex since it is composed of a [4Fe4S] cubane linked via a cysteine bridge to a 2Fe subcluster comprising the CO and CN^-^ molecules and an additional dithiomethylamine ligand^3–5^. This 2Fe subcluster needs a specific set of maturation proteins to be first assembled in the active form and then inserted into the target functional [FeFe]-hydrogenase. Three conserved proteins are involved in its biosynthesis and delivery, *i*.*e*. HydE, HydF and HydG, discovered in the unicellular green alga *Chlamydomonas reinhardtii* and then found in all microorganisms containing a [FeFe]-hydrogenase^6^. Both HydE and HydG are radical S-adenosylmethionine (SAM) proteins whereas HydF is a GTPase carrying a [4Fe4S] cluster binding motif^6^. The 3D crystal structures of all these maturases have been solved^7–10^, and several *in vitro* and cell-free experiments using purified recombinant proteins have been performed to understand their functions, allowing to propose a two-steps model that describes a H-cluster biosynthetic pathway in which a 2Fe precursor is assembled and chemically modified on a scaffold protein prior to the transfer to the apo-hydrogenase (as reviewed in refs 11 and 12). Several independent experiments indicated that HydE and HydG would be responsible for the dithiomethylamine and CO/CN^-^ biosynthesis respectively^9–17^, and that the double key role of scaffold and carrier of the H-cluster precursor is played by HydF^18–21^. Although many unresolved questions remain, the most supported model consists in the building of the synthon Fe(CO)_2_CN by HydG, with two synthons needed to synthesize a [2Fe]_H_ subcluster^16^. From the other side, a synthetic [2Fe] precursor was loaded into HydF to finally yield the active hydrogenase (HydA)^22^, suggesting a key role of HydF in the last steps of the H-cluster maturation and delivery. As a scaffold protein, HydF must efficiently interact with the two maturation partners (*i*.*e*. HydE and HydG), and with the target apo-hydrogenase itself, keeping them in close proximity in order to get a functional unit able to follow the ordered biosynthetic pathway described in the proposed two-step model. Several functional insights have been gained from the 3D structure of the apo-HydF (*i*.*e*. devoid of both GTP and FeS cluster), as well as from several spectroscopic analyses of the FeS cluster carrying protein in solution. HydF is a dimer in which each monomer is composed of three distinct domains, two with the consensus sequences for the binding of GTP (domain I) and of a [4Fe4S] cluster (domain III)^8^; the third domain (domain II) allows two monomers to associate through a large surface, giving rise to a stable, left-handed helical shaped dimer with an open and accessible surface enabling it to interact with potential partners^8^. The [4Fe4S] cluster coordination sphere of HydF has been thoroughly investigated by spectroscopic analysis of the protein in solution^19, 23–29^, which provided several clues on how the H-cluster precursor is kept in-site by the scaffold during biosynthesis and chemical modifications by HydE and HydG; however, the exact mechanism by which the mature precursor is transferred from the scaffold to the hydrogenase has not been clarified. A further unresolved issue is the specific role of the HydF GTPase moiety, which is essential for the [FeFe]-hydrogenase maturation and activation^30^. We found that the binding of GTP to HydF induces the dissociation of HydE and HydG from the scaffold^31^, suggesting that the GTPase domain could be involved in a dynamic network of interactions with the other maturases. Interestingly, the crystal structure of the apo-HydF protein showed the existence of flexible loops in this domain, which could undergo structural rearrangements upon GTP binding^8^. This could in turn have an impact on the capability of the holo-protein to interact with HydE and HydG in the maturation machinery, and to drive the proper delivery of the H-cluster precursor to the target hydrogenase.

In a previous work, we investigated the intrinsic conformational changes triggered in HydF upon GTP binding using a recombinant form comprising only the HydF domain I. Nitroxide spin labeled cysteine residues were introduced at diagnostic positions in different elements of the protein secondary structure, with the aim of monitoring large rearrangement of the structure^32^. Combining CW-EPR (electron paramagnetic resonance) and PELDOR (pulse electron-electron double resonance) spectroscopic analysis, either in the absence or in the presence of GTP, we monitored the local mobility of the spin label at the selected sites and the distance between couples of labels, respectively. We found that the binding of the nucleotide to the isolated HydF GTPase domain does not induce large conformational effects, at least at the level of the positions investigated. Instead, small changes in the distance between spin labels were observed, suggesting diffuse rearrangements upon GTP binding at the level of these structural elements^32^.

Since the presence of the other two domains may be important in producing structural constrains in HydF, by directing and/or amplifying the conformational changes induced at the GTP binding site, in the present work we used CW-EPR and PELDOR analysis by mapping the GTP-induced conformational changes along the entire HydF protein. We provide the first hint that the HydF GTPase domain functions as a molecular switch, similarly to other small GTPases^33, 34^ containing the GTP-sensitive switch regions 1 (sw1) and 2 (sw2). Strikingly, we recognized sw1 and sw2 regions in the HydF GTPase domain and showed that, upon GTP binding, the protein undergoes conformational changes which are likely instrumental in promoting HydF activity in the maturation process of hydrogenases^35, 36^.

## Results

### Design of recombinant mutants of HydF GTPase domain by *in silico* analysis

**Figure 1:**
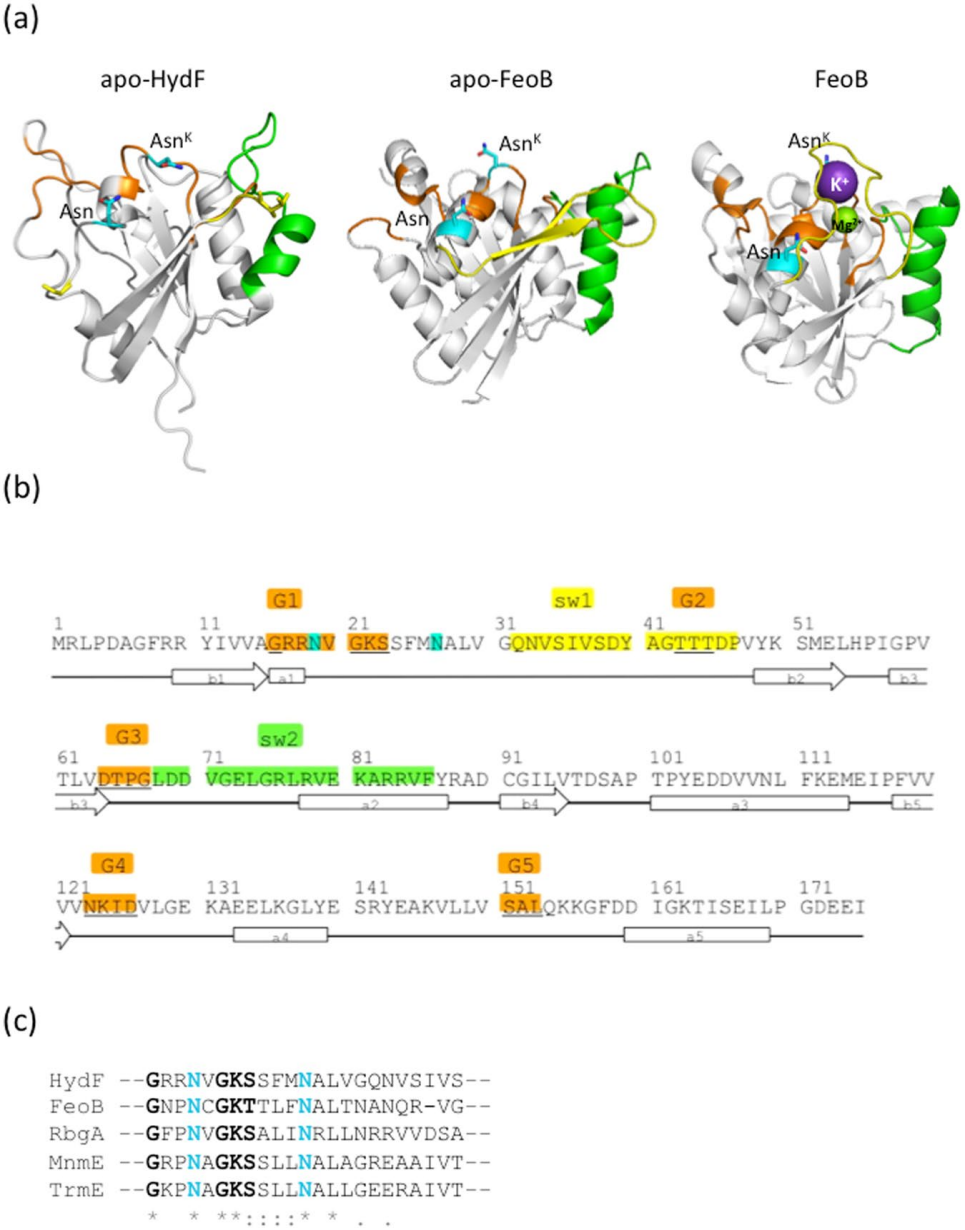
Structural features of the GTPase domain of HydF (Panel a, left. PDB ID: 3QQ5) and of the G-domain of *Streptococcus thermophilus* FeoB in the apo form (Panel a, middle. PDB ID: 3LX5) and in the holo-form binding GDP_AlF4 (Panel a, right. PDB ID: 3LX8). The magnesium atom that binds at the active site is shown as a green sphere, and the potassium atom as a violet sphere. The GDP_AlF4 ligand is not shown. GTP binding residues (orange), sw1 (yellow) sw2 (green), conserved Asn (cyan) are highlighted. The colour code is adopted from ref. 37 and maintained in the displayed structure. Note that in the structure of the GTPase domain of HydF sw1 is almost completely unresolved and consequently not displayed. Panel b: aminoacid sequence of HydF GTPase domain, with positions of G1–G4 (orange), sw1 (yellow) and sw2 (green) indicated. G5 (orange) corresponds to the less conserved G domain motif, that usually participates in recognition of the guanine base. Asparagine residues highly conserved in K^+^ activated small GTPases are marked in cyan. Secondary structure elements are also indicated. Panel c: Detail of the multiple sequence alignment (MSA) of HydF, FeoB, RbgA, MnmE and TrmE GTPase domains, generated by Clustal Omega algoritm. An *indicates positions which have a single, fully conserved residue, a: indicates conservation between groups of strongly similar properties and a. indicates conservation between groups of weakly similar properties. The complete MSA is reported in Supplementary Fig. S1. G1 motif and conserved asparagine residues are highlighted with black and cyan bold characters, respectively.

An active HydF GTPase domain is essential to produce a functional [FeFe]-hydrogenase, both *in vivo* and *in vitro* ^6, 30^. Sequence analysis indicated that the HydF GTPase domain contains the consensus motifs shared by all NTPases and essential to bind and hydrolyze GTP, *i*.*e*. the P-loop: (GRRNVGKS) and G2 to G4 loops (TTT, DTPG and NKID)^6, 30^. However, the role of GTP binding and/or hydrolysis in the H-cluster assembly is still elusive. By structural analysis, we found a similarity of the HydF folding with that of small GTPases, which are well-established regulators of several cellular functions, such as FeoB, MnmE, RbgA and TrmE. These GTPases alternate between GDP-bound and GTP-bound forms, differing by the conformations of the so-called switch 1 (sw1) and switch 2 (sw2) regions, and of other, more protein dependent, structural elements^33, 34, 37^ (see Fig. 1, panel A, where FeoB is taken as an example of the two different forms). In the HydF GTPase domain, we recognized the regions corresponding to putative sw1 and sw2, together with the GTPases consensus motifs (Fig. 1 panels A and B). Moreover, according to previous experimental evidences indicating that the HydF GTPase activity is increased in the presence of K^+^^19^, the region of HydF nucleotide-binding G1 motif (…GRR**N**VGKSSFM**N**ALV…) contains two asparagine residues, namely Asn19 and Asn27, which are highly conserved in the K^+^ activated G-proteins^37^, as indicated by a detail of the multiple sequence alignment reported in panel C of Fig. 1 (see Supplementary Fig. S1 for the complete alignment). In all reported structures of the K^+^ activated GTPases, the first conserved asparagine is a ligand to the potassium ion, which is also coordinated by three oxygen atoms from the GTP nucleotide and two backbone carbonyl groups from the sw1 region.

In order to coordinate K^+^, sw1 must adopt a particular structure in which its ‘K-loop’ lies directly over the nucleotide binding site. The GTP-bound sw1 conformation is a unique feature of the cation dependent GTPases. Moreover, in K^+^-activated GTPases the second conserved asparagine residue forms hydrogen-bonds with the backbone of sw1, and contributes in positioning it in the proper conformation. Interestingly, the putative sw1 of the HydF GTPase domain was not resolved in the X-ray structure of the apo-protein^8^, likely due to the high flexibility of this loop, which in the mentioned homologous proteins undergoes large conformational rearrangement upon GTP binding. Since the structure of HydF in the presence of either GTP or GDP is not yet available, the hypothesis of a structural analogy of its sw1 in the GTP-bound state with those of other K^+^-activated GTPase guided our experimental design aimed to detect possible rearrangements upon nucleotide binding. Panel A of Fig. 1 reports the details of the mentioned structural elements of the HydF GTPase, together with the apo- and [K^+^/Mg^2+^/GDPAlX_4_]-structures of the GTPase domain of FeoB, a membrane protein that imports Fe^2+^ ^38^, taken as reference structure of a K^+^-activated GTPase. Note that the putative sw1 region of HydF (residues 31–46) is mostly missing, since it was unresolved in the X-ray structure^8^, while the two conserved asparagines are highlighted.

As reported above, sw2 is another common protein region of the GTPases, which usually undergoes structural modification upon nucleotide binding/hydrolysis. This part is well resolved in the HydF X-ray structure and corresponds to a long loop ending with an α-helix (Fig. 1, panel A). A similar sw2 motif was found in FeoB, as clearly seen in the structural comparison. In K^+^-activated GTPases, the structure of sw2 and its rearrangement vary in a much more protein-dependent way with respect to sw1^37^. Thus, it is difficult to foresee the conformational change of this protein region upon GTP binding/hydrolysis. The same consideration holds for other protein segments, which may be involved in the specific interactions with other proteins or domains in relation to the protein function.

**Figure 2:**
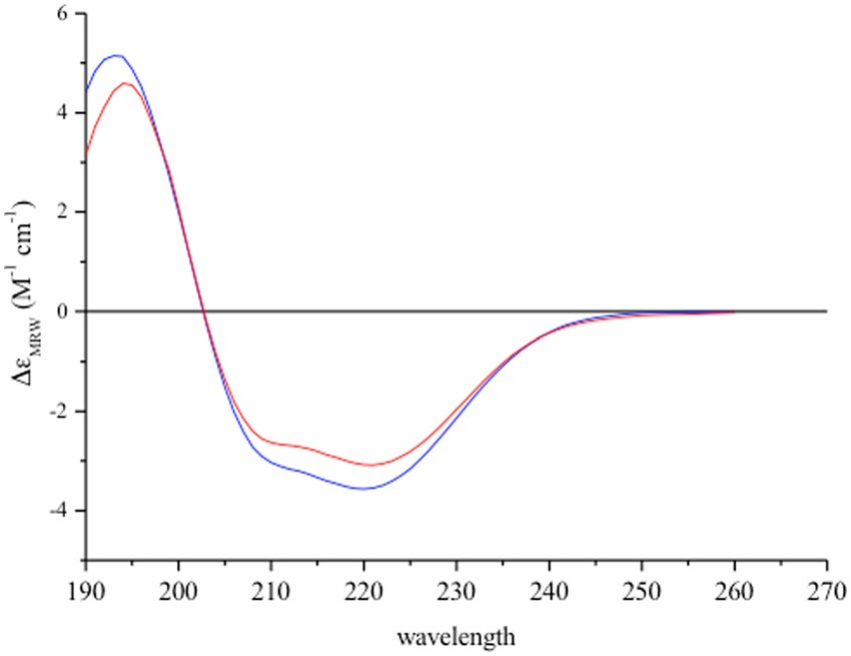
Far-UV CD spectrum of HydF taken before (blue) and immediately after (red) addition of GTP.

We obtained a first hint of a structural change induced by GTP in HydF from the circular dichroism spectra of a recombinant HydF protein expressed in *Escherichia coli* (see below), as shown in Fig. 2. The GTP binding induces a change of the secondary structure of the protein corresponding to a few percent decrease of ellipticity. The deconvolution of the CD spectra suggested a possible change of an α-helix element into β-strand and random coil traits (Supplementary Table S1).

### Heterologous expression, purification and site-directed spin-labeling (SDSL) of HydF proteins

To get insight into the specific regions undergoing the conformational changes suggested by the CD results, we made use of SDSL combined with EPR spectroscopy. This technique requires the introduction of a unique spin label that reports on localized regions of a protein^39^. All native cysteines must be eliminated in order to obtain a protein carrying a single cysteine introduced in the position of interest. This cysteine is then chemically modified with a sulfhydryl-specific EPR probe. To this end, a recombinant HydF protein was expressed in *E*. *coli* in frame with a 6His-tag at the N-terminus, as described in details in the Methods section, and purified by combining a NiNTA affinity and a gel filtration chromatography. Due to the presence of cysteine residues in wild type HydF at site 91 (GTPase domain I) and sites 302, 353 and 356 (FeS cluster binding domain III), we first substituted these native cysteines with serines by means of site-specific mutagenesis, in order to obtain a cysteine-less pseudo-wild type mutant protein. Only in one case C356 was maintained and spin labeled itself (see below). It should be noted that the removal of the cysteines in the domain III, where a [4Fe4S] cluster is bound to the holo-protein, precludes the cluster assembly; however, since the GTPase domain is not directly affected by the absence of the FeS cluster, the analysis of the cysteine-less mutants are meaningful. Moreover, the effect of the GTP binding in the CD spectrum of the cysteine-less mutant was the same as that of the recombinant wild type protein giving a comparable analysis in terms of secondary structure composition change upon nucleotide binding (see Supplementary Fig. S2 and Table S1).

**Figure 3:**
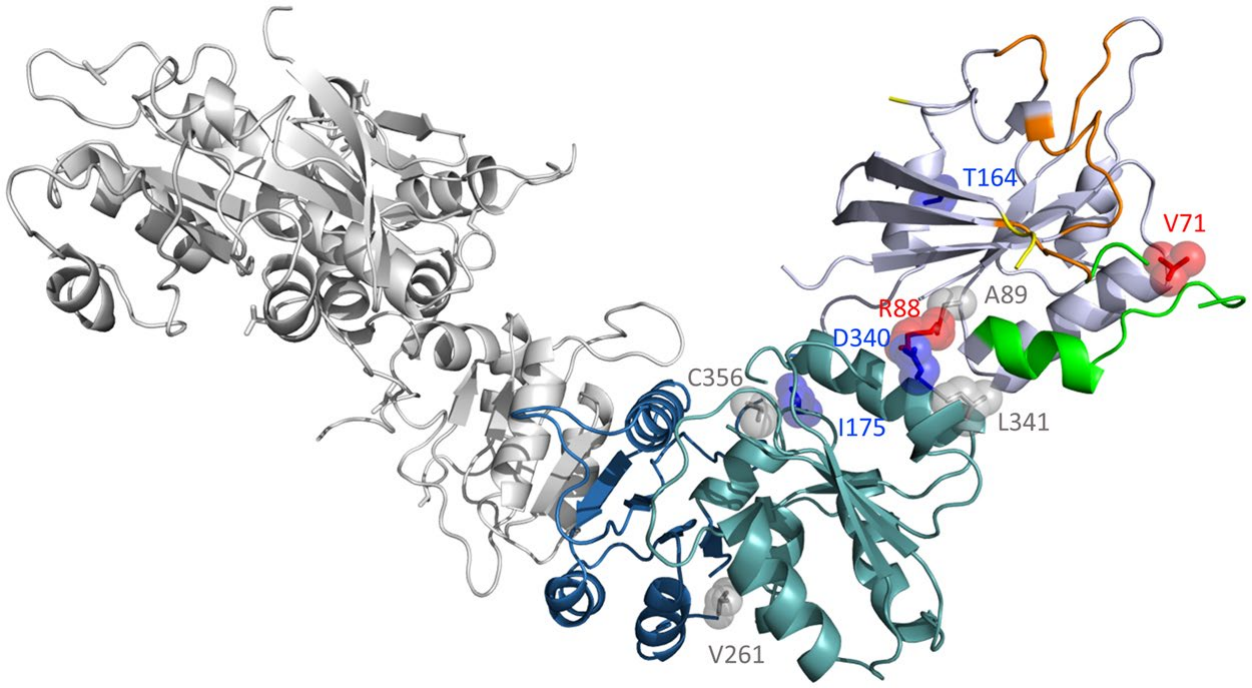
Cartoon representation of HydF monomer structure (PDB ID: 3QQ5). The GTP-binding domain is coloured in light blue violet, the dimerization domain in blue and the cluster-binding domain in teal. The P-loop is highlighted in orange, the terminal parts of sw 1 in yellow and sw 2 in green. The residues that have been mutated and labeled are indicated in the structure and coloured according to the effect on change mobility undergone upon GTP binding and detected by EPR (red, blue and grey, going from high to low effect). Positions S35, S38 and T44 are not highlighted, as they were not resolved in the crystallographic structure.

The spin labeling positions were selected based on the *in silico* analysis described above, to explore possible rearrangements of: 1) the switch regions (sw1: S35C, S38C, T44C; sw2: V71C); 2) the interface region between the GTPase domain and the catalytic domain (R88C, A89C, D340C, L341C); 3) the catalytic domain (C356); 4) the helix connecting the GTPase domain with the long loop leading to the dimerization domain (T164C); 5) the long loop itself (I175C); 6) the dimerization domain (V261C). Figure 3 reports the HydF structure with all these residues highlighted except for residues S35, S38 and T44, which belong to the unresolved loop in the X-ray structure. All single mutants were labeled using the spin label MTSSL. In some cases, 3-maleimido-proxyl (5-MSL), having a higher steric hindrance, was also used. The labeling yields, calculated by spin quantification of the EPR spectrum double integrals and comparison with those of standard solutions of the free spin labels are reported in Supplementary Table S2.

All the spin labeled mutants showed changes of the CD spectrum upon GTP binding, indicating that the introduction of the spin label was not altering the capability of the protein to adopt the nucleotide-induced structural changes.

### CW-EPR spectroscopy

**Figure 4:**
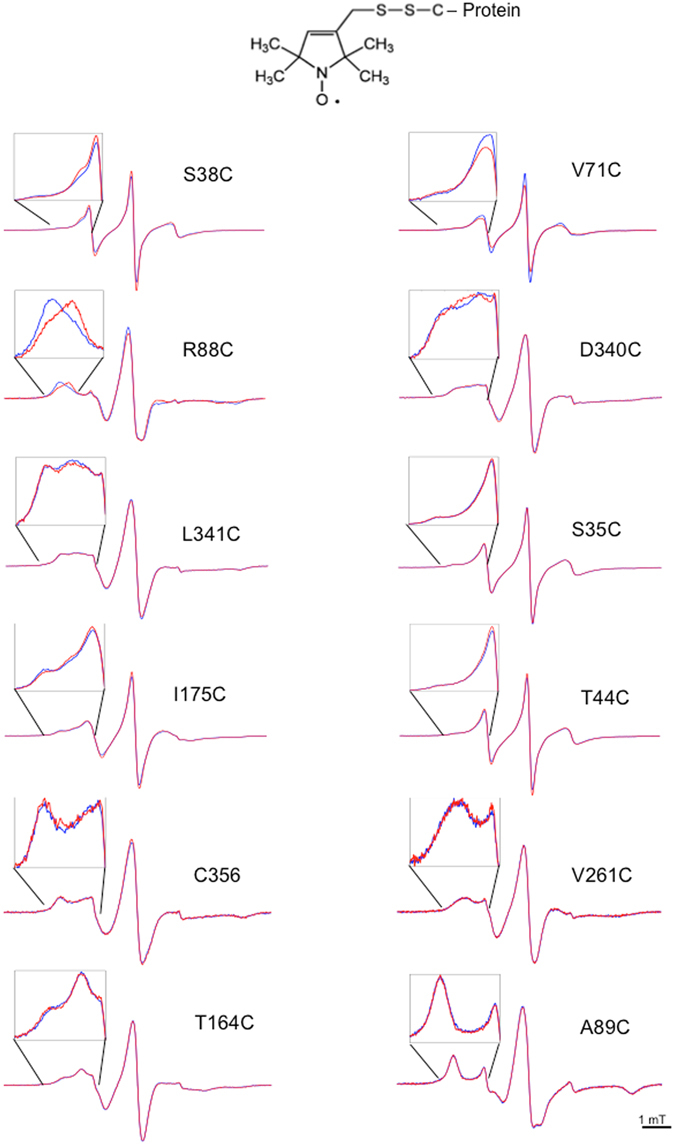
CW-EPR spectra of the 12 investigated mutants of HydF labeled with MTSSL, taken before (blue) and immediately after (red) addition of GTP. For each mutant, an enlargement of the low-field region is shown. Spectra are taken at room temperature.

**Figure 5:**
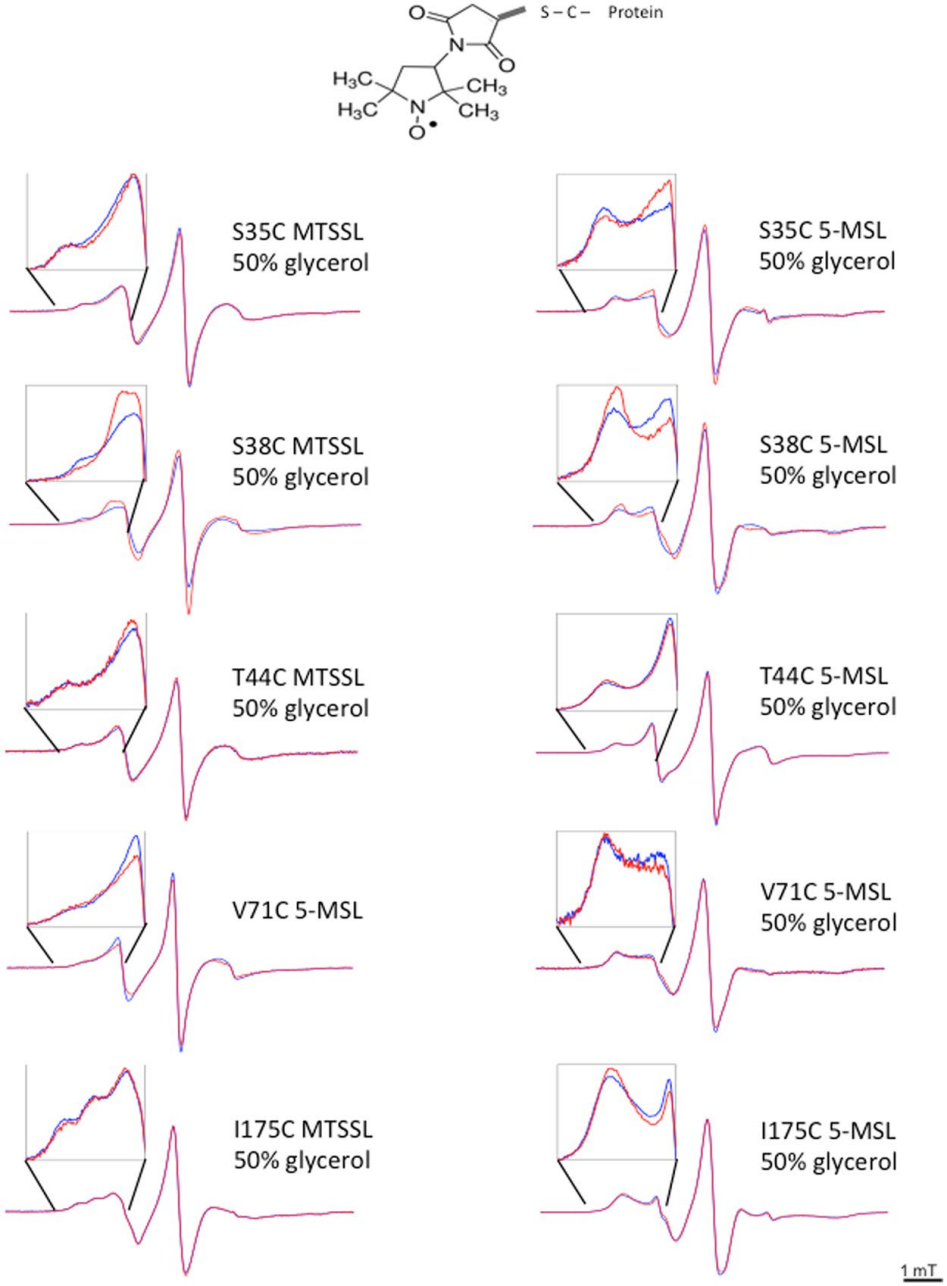
CW-EPR spectra of some HydF mutants labeled with either MTSSL (50% v/v glycerol) or 5-MTS (in the presence or in the absence of 50% v/v glycerol) taken before (blue) and immediately after (red) addition of GTP. For each mutant, an enlargement of the low-field region is shown. Spectra are taken at room temperature.

The combination of site-directed spin labeling (SDSL) and electron paramagnetic resonance (EPR) is a well-established method to determine protein dynamics and conformations^39, 40^. Following protein site-directed cysteine mutagenesis, a nitroxide spin label binds to the mutated Cys residue and reports on local dynamics, conformational dynamics of protein domains, and possibly, global protein motion. The lineshape of the EPR spectrum of a spin label reflects its mobility and is therefore sensitive to conformational changes. Highly mobile spin labels, as those on the surface of a protein, have a characteristic narrow spectrum with three sharp peaks, whereas the reduction in mobility, due to intramolecular constrains, leads to the broadening of the spectrum and/or the appearance of an additional peak in the low field region^41^. We recorded the EPR spectra of purified HydF proteins individually spin-labeled with MTSSL at the 12 different positions mentioned above, and looked for mobility changes upon GTP addition (Fig. 4). In the majority of the explored sites, the spin probe exhibited multiple motional states, indicating that either the side chains of the probes may have different motional states and/or the protein backbone may assume different conformations. Notable effects upon GTP binding were detected at position 38, belonging to sw1, 71, belonging the sw2, 88, corresponding to the terminal part of sw2 and to the interface region of GTPase doman with the catalytic domain. In some cases, conformational changes were better evidenced by using 3-maleimido-proxyl (5-MSL) (Fig. 5). While 5-MSL is rigidly attached to the protein, providing information on the rotation of whole structural elements of the labeled protein, MTSSL is bound by a more flexible linkage and describes better the local environment of the target residue in the protein structure. It can be seen that two dynamic components are present in the EPR spectrum of 5-MSL labeled protein at position 38, while in the corresponding MTSSL labeled protein three components are contributing to the spectrum. The differences are even more pronounced if glycerol is added to the buffer solution (50% v/v) to increase the viscosity of the medium and thus lengthen the correlation time of the motions. The changes induced by GTP binding lead to a redistribution of the different components. Also in the case of position 71, 5-MSL is more affected by the nucleotide binding compared to MTSSL.

Minor, but still detectable, mobility changes were observed at positions 35 and 44 (sw1), 340 (catalytic domain, region facing residue 88), 164 (α-helix of GTPase domain close to the loop connecting the dimerization domain), and 175 (belonging to the long loop connecting the dimerization domain) (Fig. 5). For these positions, the effects were strongly dependent on different conditions such as spin label structure and/or addition of glycerol. In the case of S35 and T44 changes on the EPR lineshapes are observed in particular when 5-MSL is used as spin label in the presence of glycerol, that is when the mobility is reduced. In these conditions changes are detected which, although small, are reproducible.

Finally, very little or no effects were detected at position 341 (catalytic domain, region facing residue 88), 356 (catalytic domain, position corresponding to [4Fe4S] cluster binding in the functional protein), and 261 (dimerization domain).

**Figure 6:**
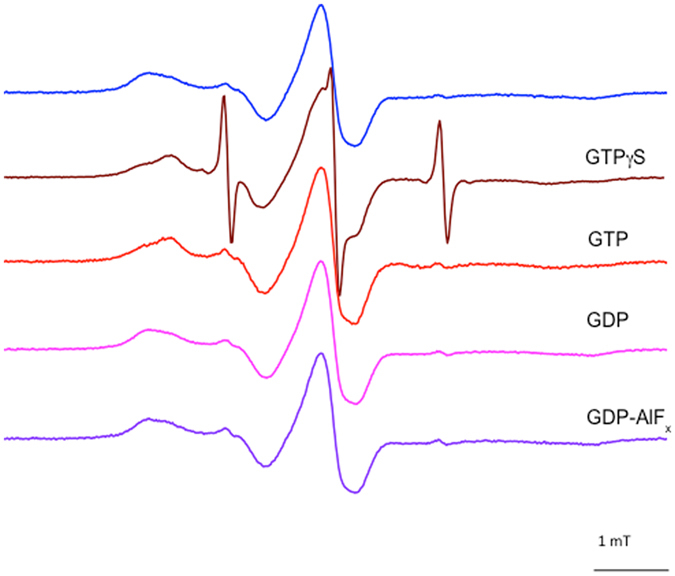
Effects of different nucleotides binding on the CW-EPR spectrum of labeled R88C: absence of nucleotides (blue), in the presence of non-hydrolysable GTP analogue (brown), GTP (red), GDP (magenta) and GDP-AlF_x_ (violet).

To better characterize the role played by the nucleotides in determining the conformation of HydF, the effects of GDP, GDP-AlF_4_ (a transition-state analogue) and GTPγS (a non-hydrolysable GTP analogue) were also explored. The results are shown in Fig. 6 for the MTSSL spin labeled R88C mutant, which was the one showing the clearest mobility change upon GTP binding. No spectral changes were observed upon addition of GDP and GDP-AlF_4_, while GTPγS induced the same effect as GTP (note that in the presence of GTPγS the spectrum showed a high fraction of free spin label, due to the release of MTSSL by reaction of the spin labeled protein with GTPγS itself).

**Figure 7:**
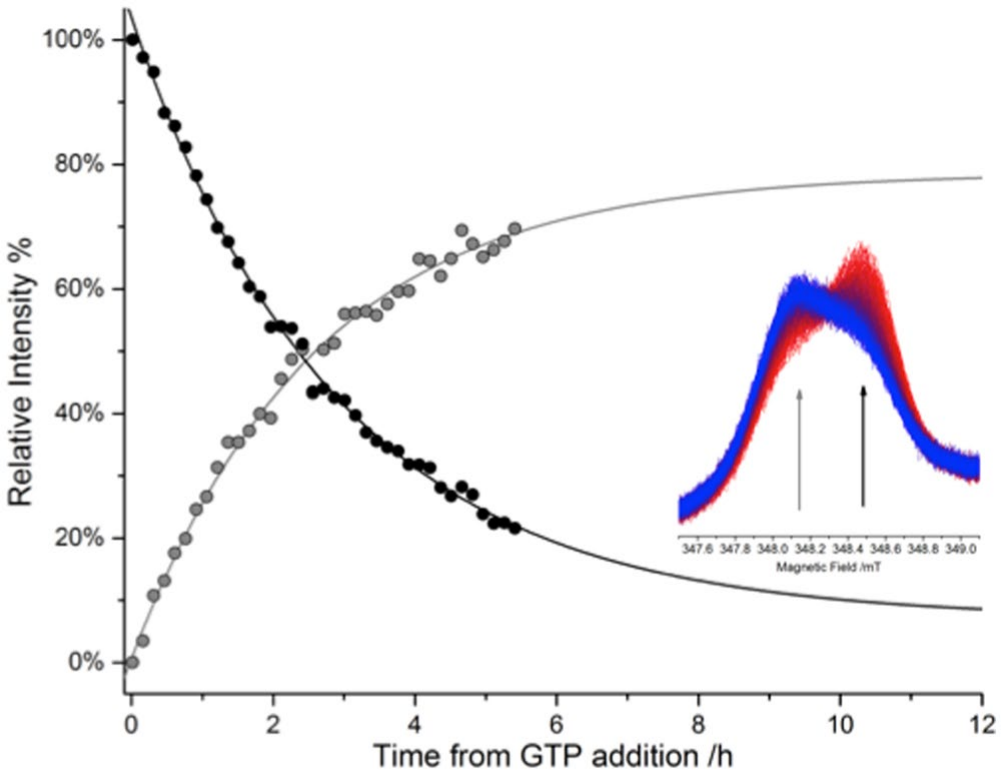
Curves of the relaxation to the initial state following addition of GTP (t = 0 h), detected as change of the EPR signal of spin label at site 88, at the field positions indicated by arrows in the inset. The dots represent respectively the percentage of recovery of the broader spectral component (grey dots) and of decay of the narrower spectral component (black). In the inset, the zoom of the EPR spectra (red t = 0 h; blue t = 5.5 h) with the positions used to obtain the kinetics shown by arrows.

As expected, the conformational changes were reversible once the GTP was hydrolysed and left the protein binding site. Interestingly, the relaxation to the initial state was very slow compared to the kinetics of hydrolysis. Indeed, while the hydrolysis of the nucleotide occurs in minutes^19, 31^, detection of the EPR spectrum at different delay times after the GTP addition showed that only after several hours the spectrum returned to the lineshape preceding the nucleotide addition (see Fig. 7).

A reported above, the HydF GTPase domain contains the conserved residues of K^+^-activated GTPase. Accordingly, it was previously reported that potassium largely increases the hydrolysis rate^19^. Thus, we performed the EPR experiment on R88C also in a buffer solution without K^+^. The analysis of these spectra indicates that the absence of K^+^ does not preclude the conformational change induced by GTP addition, however the extent of the change is reduced (Supplementary Fig. S3).

### PELDOR

Since HydF adopts a dimeric structure^8^, with the aim to map possible large conformational changes induced by the GTP binding at the level of this dimeric structure, we also performed Pulse Electron DOuble Resonance (PELDOR, also known as DEER) experiments^42^. It is well known that this pulse EPR technique, based on the measure of dipole-dipole interaction between unpaired electron spins, has become the most widely used method for measuring distances between electron spins in (bio)macromolecules. The V261C HydF mutant was chosen to perform intra-dimer distance measurements because, based on the X-ray structure, the expected distance between spin labels belonging to the two moieties composing the dimer is 3.5 nm, which is in the suitable range of reliable distances measured by PELDOR. Moreover, according to the X-ray structure, residue 261 is located in a portion of the dimerization domain, that does not interfere with the folding of the β-sheet forming the dimeric structure of HydF, thus representing a good choice to detect conformational changes induced by GTP at the level of the dimer structure.

**Figure 8:**
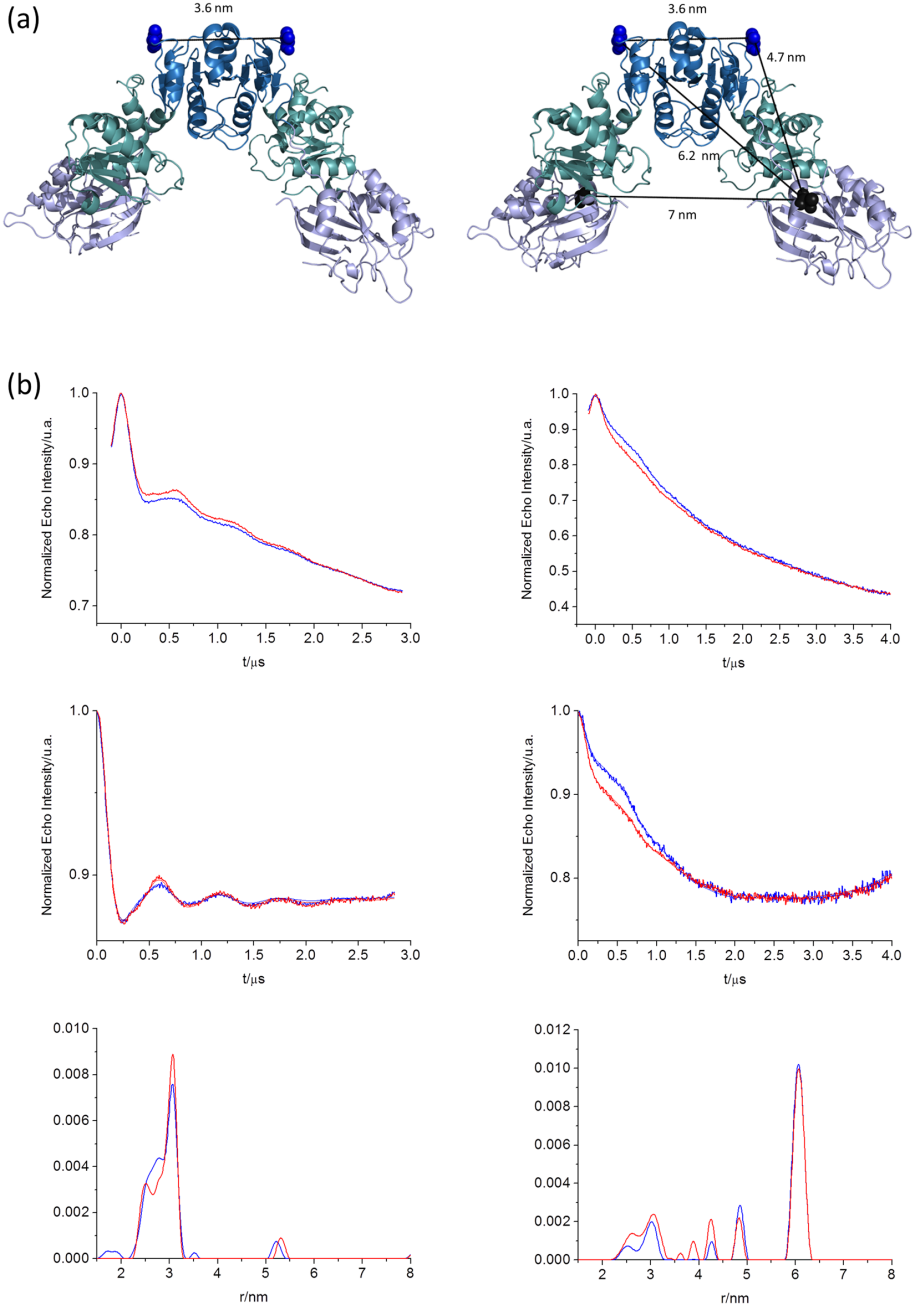
PELDOR of spin labeled V261C and V26C-T164C with HydF dimer structure representation. (Panel a) Cartoon representation of HydF dimer structure with the indication of the distance between the residues V261 (left) and between residues V261 (blue) and T164 (black) (right). (Panel b) PELDOR data of mutant V261C (left) and V26C-T164C (right), in the absence (blue) and in the presence (red) of GTP before (upper panels) and after (middle panels) background correction, Tikhonov-derived distance distributions in the absence (blue) and in the presence (red) of the nucleotide, are shown in the bottom panels.

The spectra of samples frozen in the absence of GTP and immediately after its addition are reported in Fig. 8, together with the data analysis. The very good signal to noise ratio allowed us to obtain a reliable measure of the effects. Tikhonov-derived distance distributions provided main values, that correspond, roughly, to those expected on the basis of the X-ray structure of the apo-HydF protein (3.5 nm), confirming the dimer structure of the protein in solution. When GTP was added, some differences were detected. The distance distribution showed that about 25% of the shortest distance (2.8 nm) is converted into the longer (3.0 nm) in the presence of GTP. This is a clear indication of a rearrangement occurring in a protein region far from the nucleotide binding site. It does not correspond to a dramatic reassembly of the dimer, however the protein region around V216 clearly “feels” the switch triggered by the GTPase domain. We also performed the PELDOR experiments in a double spin labeled mutant (V261C-T164C) having an expected intra-monomer distance of 4.5 nm and estimated inter-monomer distances (261–164 and 164–164) 6.2 and 7.0 nm, respectively. The spectra, reported in Fig. 8, show also in this case emerging differences when GTP was added. Although quantitative analysis of multispin systems (four spins in this double labeled mutant) is quite complicated^43^, it seems clear that such differences are present not only in the region corresponding to the 261-261 distance, as for the single labeled mutant V261C, but also at the level of the other inter- and intra- monomer distances. Validation of the distance analysis performed with DeerAnalysis2015 is reported in Supplementary Fig. S4.

## Discussion

The sequence homology of the HydF GTPase domain with those of proteins belonging to the K^+^ activated GTPase family is a strong indication of its possible role as a molecular switch. The suggested double function of HydF as scaffold and carrier of the 2Fe unit of the [FeFe]-hydrogenase (HydA) H-cluster precursor may be facilitated by conformational changes of the protein during the cycle of interaction with HydG, HydE and/or HydA. Shepard and coworkers previously showed that the HydF-dependent GTP hydrolysis *in vitro* increases in the presence of HydE or HydG^19^, suggesting the existence of a HydF GTPase domain function/structure relationship driving the interactions of this scaffold with the other two maturases. More recently, Vallese *et al*. showed, based on Surface Plasmon Resonance experiments performed by injecting the nucleotide during the step of HydE and HydG dissociation from HydF, that the binding of GTP increases the dissociation rate^31^. This could be related to the maturation mechanism by which the displacement of an interaction partner from the scaffold occurs, allowing subsequent association of a different protein. Thus, it seems likely that a conformational switch due to the GTP binding to HydF is responsible for a fast release of the other two maturases.

With the aim to prove the occurrence of conformational changes of HydF upon GTP binding, we first used CD spectroscopy. The addition of GTP induced a clear change of the secondary structure, reflected in the CD spectrum. However, the technique is not very sensitive to the rearrangement of unstructured regions, such as loops or random portions of the protein. Therefore, to get further insight into the protein regions undergoing structural rearrangement, we used SDSL EPR spectroscopy, which, with the introduction of a unique spin label at specific sites, reports on localized regions of a protein. If the nucleotide binding induces some changes in the protein structure, the lineshape of the EPR spectrum of a spin label is expected to change, when the probe is located at a site involved in/close to the rearrangement. A highly mobile spin label on the surface of a protein has a characteristic narrow spectrum with sharp peaks, whereas the reduction in its mobility due to intramolecular constrains or “trapping” in a protein-protein interaction interface is reflected in the broadening of the spectrum.

To provide adequate coverage of the protein structure analysis by EPR, we expressed and purified 12 cysteine single mutants, modified them with spin labels and compared their EPR spectra taken before and after GTP addition. In this way, we were able to obtain information concerning different protein regions. Most of the changes detected in the EPR spectra of HydF are not as dramatic as one would expect for a large protein reassembly and reshaping. The 12 positions probed by our experiments, however, are indicative of structural changes taking place at different extent depending on the protein region.

Significant effects are found at the level of putative sw1 and sw2, in particular at positions 38 and 71. Looking at the structural analogies common to all the known K^+^ dependent GTPases at the level of sw1, it seems likely that in HydF, after GTP addition, residue 38 could become very close to the K^+^ binding region, starting from an extended loop far from this site. This would be consistent with the observed reduced mobility of the backbone, as revealed by the 5-MSL spin label. The MTSSL spin probe at the same positions has a more complicated behaviour, described by three components. The most immobilized component undergoes a reduction in intensity as well as the most mobile, while the intermediate regime component gains intensity. This suggests that MTSSL, having higher flexibility compared to the 5-MSL, may adopt different local conformations. Since both spin probes reveal a redistribution of the components of the EPR spectrum upon GTP binding, a significant structural change leading to many local effects is clearly taking place. Residues 35 and 44, which are very close to 38, undergo similar changes in terms of redistribution of components, although less pronounced. Changes of EPR lineshapes are observed for these two residues when 5-MSL is used as spin label and in the presence of glycerol, when their mobility is reduced. Looking at the spectra of the two proteins labelled with MTSSL it appears that residues 35 and 44 are both characterized by relatively high intrinsic mobility either in the presence or in the absence of GTP. For residue 44 this was expected on the basis of a possible structural analogy of HydF with FeoB (Fig. 1) in the apo form. In that protein, when sw1 undergoes the large conformational change this residue moves from a loop position towards another, although different, loop position. Thus, its intrinsic mobility is expected to undergo only little changes. On the contrary, position 35 was expected to behave differently starting from the equivalent position in apoFeoB structure, that is a β-strand which rearranges into a loop interacting with the K^+^ binding site upon GTP binding. However, it is worth noting that an unresolved sw1 region was found in the X-ray structure of apo MnmE [PDB:3gee], and a long loop was characterizing the sw1 in the X-ray structure of apoTrmE [PDB;1xzp]. Both these proteins are members of the K^+^ GTPase family and show the conserved structure of sw1 which characterize the family in the GTP-bound state. Thus, it is likely that also in HydF sw1 is characterized by a loop in the apo form which rearranges upon binding of the nucleotide into another loop, close to the K^+^ site.

A clear structural rearrangement is detected at position 71, in the putative sw2 region, which, according to the X-ray structure, adopts a loop conformation in the apo-form of the protein. The EPR results indicate an immobilization of this residue upon GTP binding. Taken together, all the effects relative to residues 35, 44, 38 and 71 strongly corroborate the hypothesis of a molecular switch role for the GTPase domain of HydF.

As observed for other GTPases, the conformational changes may extend to additional portions of the molecular structure, more protein dependent, which could be relevant for specific protein-protein interactions and/or protein-ligand assembly [ref. 37 and refs therein]. Since HydF interacts with HydE/HydG and is believed to act as a scaffold for the assembly and delivery of the 2Fe unit of the H-cluster, it is well possible that the GTP binding either produces some effects in regions of the protein involved in the interaction with the other maturation proteins or induces modification in the catalytic domain of HydF, where the cluster precursor is bound and processed. In this respect, the extended change on spin mobility measured by EPR at the position 88 is very interesting, since this residue is located at the interface between the GTPase domain and the catalytic domain. The increase of mobility detected at this site after GTP addition may be related to a larger separation of the two domains induced by the nucleotide binding. A confirmation of this interfacial change is also proven by the effect experienced by the spin probe at site 340 belonging to the catalytic domain and facing residue 88. The effect at site 88 was not observed in our previous work on isolated GTPase domain^32^, because the spin probe was completely exposed to the solvent due to the absence of domain III.

The structure and length of the sw2 region are more heterogeneous in the GTPases compared to those of sw1. The conformational change in sw2 upon nucleotide binding differs among distinct GTPases, ranging from small rearrangements, such as in Ras^44^, to a major reorientation of helix a2, as in EF-Tu^45^. Interestingly, in NFeoBLp, sw2 includes a long loop region (10–14 residues) and a helix (a2). The unique location of a2 between the nucleotide-binding site and the GDI domain of NFeoBLp suggested for this helix a function of relay element transmitting the signal induced by nucleotide binding to the GDI and transmembrane domains^46^. In this way, nucleotide binding to the G domain in FeoB regulates ferrous iron uptake across the membrane. The 3D structure of HydF indicates that sw2 contains a long loop constituted by 14 residues followed also by a helix (a2). R88 is located at the base of a2, facing domain III. Thus, an effect similar to that observed in FeoB proteins could take place in HydF with the conformational change of sw2 upon nucleotide binding transmitted to the catalytic domain via a2, as suggested by the change in mobility experienced by the spin probe at site 340. In this respect, it is worth noting that the EPR spectrum of HydF [4Fe4S] cluster was found to be sensitive to GTP^19^. We did not observe changes in the spin label mobility at position 356 where a Cys ligand of the [4Fe4S] cluster is present in the wild type protein, however the lack of the cluster in the recombinant mutant protein may alter the response of the spin label at this site due to the absence of structural constrains imposed by the cluster itself in the holo-protein.

HydF is characterized by a dimerization domain, which is directly connected to the GTPase domain through a long loop (see Fig. 2). Similar long loops connecting different domains and undergoing structural rearrangements upon nucleotide binding are found, for instance, in the K^+^-dependent GTPases MnmE and TrmE^47, 48^. To explore the response of the loop to the GTP binding in HydF, we considered the spin label at position 175. Interestingly, we found that the spin label bound at this site, although far away from the GTP binding site, undergoes a detectable change in the mobility upon nucleotide binding. We also monitored the possible effects in the dimerization domain by SDSL at site 261 and measuring the 261-261 inter-monomer distance by PELDOR. Clear effects were found on the order of few Å displacement upon GTP binding, showing that the changes occurring in the GTPase domain are felt by distant residues belonging to the dimerization domain as well. The effects induced on the dimer structure were further confirmed by the PELDOR analysis of the double mutant T164-V261.

Proteins acting as GTPase switches show conformational changes, induced by a cycle of GTP hydrolysis, with different mechanisms. Changes in protein forms can be promoted either by GDP binding or by GTP binding/hydrolysis^34^. Thus, it was of primary importance to investigate the effects of the different nucleotides. The addition of GDP and GDP-AlF_4_ to the protein did not produce any observable effect in the EPR spectra, while GTPγS induced the same effect as GTP, strengthening the hypothesis that the trigger of the conformational change is given by the binding of the nucleotide rather than by its hydrolysis. Thus, as common for many other GTPases, the conformation of HydF in the presence of GDP is the same as that of the apo-protein. From the time evolution of the EPR spectra after the addition of GTP, we found that the return to the apo-conformation is very slow compared to the kinetic of hydrolysis of GTP. This inertia could be due to the absence of some cellular effector in our *in vitro* experiments compared to the *in vivo* conditions, as observed for a number of GTPases needing effectors to perform the GTP/GDP cycle^34^, but could even be functional to generate a rest time for the switch, allowing other steps of the maturation process to take place. This would fit with the previously proposed stepwise model of the H-cluster assembly on the HydF scaffold^11, 12^.

Functional and sequence analysis clearly indicate that HydF is a K^+^ activated GTPase^19^. Our EPR experiments show that the absence of K^+^ does not preclude the conformational change induced by GTP binding, however its presence favours the switch of the structure. According to the known structures of the K^+^ activated GTPases, the cation contributes to the coordination of the sw1 upon nucleotide binding, thus the observed effect in HydF is in agreement with the rearrangement of sw1 and with the contribution to the stabilization of the switched conformation.

Most of the sites investigated by SDSL EPR reveal the presence of more than one conformation, both in the apo- and in the GTP-bound state. We never observed a complete conversion of the EPR spectrum from one form to another, even in large excess of either GTP or GTPγS. For instance, in the case of R88C the change in the lineshape of the EPR spectrum induced by GTP can be assigned to a 30% population shift between the two components needed for the simulation of the spectra (Supplementary Fig. S5 and Table S3). This is in agreement with the results we obtained from Isothermal Titration Calorimetry (ITC) experiments (reported in Supplementary Fig. S6 and Table S4), showing that only 60% of the wild type recombinant HydF is able to bind GTP. There are different possible explanations for this experimental evidence: a) a percentage of protein is in a misfolded conformation, therefore only a certain amount of protein is sensitive to the nucleotide binding; b) HydF is always present in an equilibrium of different conformations and the GTP binding just shifts the equilibrium among different forms. The presence of some unknown effectors *in vivo*, might restrict the conformational space of the protein generating a limited number of conformations compared to the *in vitro* conditions adopted in this work. In this respect, it is interesting to note that in cell-free experiments an addition of cellular extract is always necessary to allow the [FeFe]-hydrogenase maturation process to take place^49^. Although it is difficult at this stage to discriminate between the a) and b) possibilities, the main result remains, showing that the GTPase domain of HydF may undergo conformational changes upon GTP binding. The presence of a correct cluster assembly and/or the interaction with other unknown effectors/maturases may well enhance the differences between energies of the conformations that we have detected shifting the equilibrium between different forms, which are however intrinsically determined by the nucleotide binding. EPR experiments will be performed in the near future by using unnatural aminoacids carrying a spin label to avoid the substitution of native cysteine residues and allow refolding of the protein in the presence of the [4F-4S] cluster in the catalytic domain, with the aim of studying the influence of the cluster presence on the conformational equilibrium shift induced by GTP.

## Conclusions

In the present work, we recognized for the first time the analogies of HydF with the K^+^ dependent GTPases, in terms of sw1 and sw2 regions and conserved Asn residues, and established that the GTPase domain is a switch undergoing significant structural modifications upon GTP binding, as in other members of the same family. Starting from this discovery, it will be possible to model the sw1 in the GTP bound state of HydF, an important structural information which was missing also in the X-ray structure of the apo-protein.

Although the predicted cation-dependent GTPases from various superfamilies (TEES, Obg-HflX, and YqeH-like) are all involved in ribosome biogenesis, exceptions are reported. For instance, FeoB is a membrane protein that imports Fe^2+^ ^38^ and MnmE modifies tRNA^50^. Thus, HydF may represent an additional K^+^-activated GTPase with a novel function, showing that the K^+^ GTPases may have a larger spread of functions than supposed before.

We have provided evidence that EPR is a suitable technique to follow the changes induced by GTP binding and hydrolysis in HydF and monitor the states of nucleotide cycle. The effects monitored at different protein sites, by using SDSL-EPR techniques, showed that the structural changes upon GTP binding in HydF are diffuse, and indicate that not only the GTPase domain but the whole protein undergoes conformational rearrangements. This is in agreement with previous data suggesting that GTP alters the EPR signal of the reduced [4Fe4S] cluster of HydF, and facilitates the dissociation of HydE and HydG from HydF. The interaction areas between the two maturation proteins and HydF are not known, however they likely involve extended protein regions not only sw1 and 2. Thus, the diffuse conformational changes detected in HydF upon GTP binding may well be functional to a variation of interaction with the other maturases. Experiments to measure these effects in the HydF-HydG and HydF-HydE spin labeled complexes will help to confirm this hypothesis.

As a final remark, it is worth noting that a GTP-dependent step in the maturation process is found also in [NiFe]-hydrogenases. The maturase HypB is a metal-binding GTPase involved in this step^51^, which is essential for hydrogenase maturation/activation. Size exclusion chromatography and cross-linking studies demonstrated that the binding of GTP triggers the dimerization of HypB. The HypB GTP-dependent dimerization facilitates nickel delivery to hydrogenase by loading nickel to the metal-binding site at the dimeric interface. Thus, a GTPase-dependent molecular switch may be a common strategy adopted by the different hydrogenases in the assembly of their complex metal active sites.

## Methods

### Heterologous expression and purification of HydF proteins

The *Thermotoga neapolitana hydF* gene was isolated from purified genomic DNA by PCR amplification and subcloned in frame with a 6His-tag sequence at the N-terminus in a pET-15b vector (from Novagen®), as described in ref. 8. Site-directed mutagenesis of the *hydF* at selected sites was performed with the QuickChange® II Site-Directed Mutagenesis Kit (from Agilent Technologies), using as template *pET-15b*/*hydF* recombinant plasmid and the couples of primers listed in Supplementary (Table S5). The sequence of each mutant *hydF* gene was confirmed by DNA sequencing (at GATC Biotech, Germany). *E*. *coli* Rosetta (DE3) cells were transformed with the obtained plasmids and positive clones selected by antibiotic resistance. The expression of the wild type and mutant 6His-tagged HydF proteins was induced by adding 1 mM isopropyl-β-thiogalactopyranoside (IPTG) in LB medium and incubating the cells at 30 °C overnight. Proteins were purified starting from 0, 5 to 1 L cultures and combining affinity chromatography and gel filtration. Briefly, cells were harvested by centrifugation, resuspended in lysis buffer (25 mM Tris-HCl pH 8.0, 200 mM KCl supplemented with protease inhibitors 1 µg/ml pepstatin A, 1 µg/ml leupeptin, 1 µg/ml antipain, 1 mM PMSF) and lysed by French press. The supernatant fractions were isolated from cell debris by centrifugation and the proteins purified to homogeneity by a nickel affinity chromatography (HIS-Select^®^ Nickel Affinity Gel, from Sigma-Aldrich) and a gel filtration chromatography using a Superdex 200 GL 10 300 column (from GE Healthcare), equilibrated in a buffer containing 25 mM Tris-HCl pH 8.0, 200 mM KCl, and 1 mM MgCl_2_ (final buffer). To estimate the molecular weight of the analyzed samples, the column was equilibrated in the same buffer and calibrated with the standards thyroglobulin (669 KDa), ferritin (440 KDa), β-amylase (200 KDa), bovine serum albumin (67 KDa), carbonic anhydrase (29 KDa) and cytochrome *c* (12 KDa). The eluted fractions containing the HydF dimer were finally pooled together and concentrated by centrifugal filters (Amicon Ultra Centrifugal Filter, 10000 NMWL, from Merck Millipore) to a volume suitable for spectroscopic analysis (see below), giving rise to a final concentration up to 600 µM, as determined spectroscopically using ε_280nm_ = 26360 M^−1^cm^−1^. Purified proteins were analyzed by 12% SDS-PAGE.

### Isothermal Titration Calorimetry (ITC)

ITC measurements were carried out at 25 °C on a MicroCal OMEGA ultrasensitive titration calorimeter. The titrant and sample solutions were made from the same stock buffer solution (25 mM Tris-HCl pH 8.0, 200 mM KCl, and 1 mM MgCl_2_), and both solutions were thoroughly degassed before each titration. The solution (75 μM wild type HydF protein) in the cell was stirred at 200 rpm to ensure rapid mixing. Typically, 7 μL of titrant (500 mM either GTP or GTPγS) were delivered every 10 s with an adequate interval (4 min) between injections to allow complete equilibration. Titrations continued until no further complex formation following addition of excess titrant was detected. A background titration, consisting of identical titrant solution and buffer solution in the sample cell, was subtracted to account for heat of dilution. The data were collected automatically and then analyzed by the Windows-based Origin software package supplied by MicroCal. A one-site binding model was used.

### Circular Dichroism (CD)

CD measurements were performed with a Jasco J-810 spectropolarimeter. Far-UV CD spectra were collected using cells of 0.1 cm path-length. Data were acquired at a scan speed of 20 nm/min and at least three scans were averaged. Proteins were used at a concentration of 5 μM (0.2 mg/ml), in a 0.5 mM Tris-HCl buffer, pH 8.0, containing 4 mM KCl and 20 μM MgCl_2_. Measurements in the presence of GTP were performed in the same samples analyzed in the absence of the nucleotide, adding 1 μL of GTP to a final concentration of 250 μM in 400 μL of total volume. Experiments were performed at 25 °C using a thermostated Jasco PTC-423 Peltier Cell Holder connected to a Jasco PTC-423S Peltier Controller. The secondary structure content of HydF was calculated using the CD spectrum deconvolution software CDNN^51^. This software calculates the secondary structure by comparison with a CD database of known protein structures.

### CW-EPR

Samples for EPR labeled with either MTSSL or 5-MSL were obtained by adding to the purified protein (at a concentration of about 150 μM) a fivefold molar excess of spin label, either MTSSL or 5-MSL, dissolved in DMSO and ethanol respectively, and incubating the protein at 4 °C overnight in the dark. Excess of non-ligated spin label was removed from the protein by several cycles of dilution with final buffer (25 mM Tris-HCl, pH 8.0, 200 mM KCl and 1 mM MgCl_2_), and concentration by centrifugal filters. Twenty microliters of each sample, with a protein concentration of about 600 µM (in 25 mM Tris-HCl, pH 8.0, 200 mM KCl, and 1 mM MgCl_2_) were loaded into quartz capillaries with 0.6 mm ID and 0.84 mm OD. In GTP binding experiments, GTP (up to 10 mM) was added to the samples and EPR measurements were performed immediately after the addition. EPR spectra were collected at room temperature (298 K) on an Elexsys E580-X-band spectrometer (Bruker) using a Super High Sensitivity Probehead cavity. The field modulation frequency was set at 100 kHz, with a field-modulation amplitude of 0.5 G and the microwave power was 6.4 mW. Time constant was set at 40.96 ns and conversion time at 81.92 ms; data collection was carried out acquiring 1024 points. The center of the field was set to 351 mT and the sweep width to 10 mT. Simulations of the CW-EPR spectra were performed using a program based on the stochastic Liouville equation and adopting the MOMD model as standard for spin-labeled proteins^52^. The overall rotational correlation time of the HydF dimer was estimated using the program by Zerbetto *et al*.^53^. Details are reported in Supplementary information.

### Pulse ELectron DOuble resonance (PELDOR)

Samples were exchanged with deuterated buffer. Deuterated glycerol (33% v/v) was also added to the samples before freezing. The final protein concentration was about 150 µM for all the samples. In the nucleotide binding experiments, GTP was added to a 10 mM final concentration. All the samples, loaded into quartz capillaries with 1.1 mm ID and 1.6 mm OD, were quickly frozen. Q-band pulse EPR experiments were performed with the same EPR spectrometer used for CW-EPR (Elexsys E580) equipped with a Bruker EN 5107D2 resonator (microwave frequency = 33.86 GHz) and an Oxford CF935 cryostat. The measurements were performed at a temperature of 50 K. A standard four pulse sequence was applied; the microwave power was adjusted to obtain an observer sequence of 28/56/56 ns and a pump pulse of 56 ns. The difference between the pump and observer frequency was set to 80 MHz. A two-step phase cycle was applied for base-line correction, while deuterium nuclear modulations were suppressed using an 8 step τ cycle from a 180 ns starting value with 56 ns increment steps. Data on each sample were collected for about 15 hours. Distance distributions were extracted from PELDOR traces by using DeerAnalysis2015^54^.

## Electronic supplementary material

Supplementary information
