# Supplementary material for: Identifying conformational changes with site-directed spin labeling reveals that the GTPase domain of HydF is a molecular switch
Source: Sci Rep. 2017 May 10;7:1714. doi: 10.1038/s41598-017-01886-y (PMC5431965; doi:10.1038/s41598-017-01886-y)
Supplement: Supplementary file 1 — Supplementary information [file 41598_2017_1886_MOESM1_ESM.doc]

**Supplementary Information**

**Identifying conformational changes with site-directed spin labeling reveals that the GTPase domain of HydF is a molecular switch.**

Laura Galazzo^b^#, Lorenzo Maso^a^#, Edith De Rosa^a^, Marco Bortolus^b^, Davide Doni ^b^ Laura Acquasaliente^c^, Vincenzo De Filippis^c^, Paola Costantini^a^* and Donatella Carbonera^b^*

*^a^Department of Biology, University of Padova, Viale G. Colombo 3, 35131 Padova, Italy*

*^b^Department of Chemical Sciences, University of Padova, Via F. Marzolo 1, 35131 Padova, Italy* *^c^Department of Pharmaceutical and Pharmacological Sciences, University of Padova, Via F. Marzolo 5, 35131 Padova, Italy*

**Multiple sequence alignment (MSA) of HydF, FeoB, RbgA, MnmE and TrmE GTPase domain**

**CD spectra analysis of HydF WT and cysteine-less mutant**

Comparison of the CD spectra of HydF WT (spectra reported in the main text) and cysteine-less mutant (Figure S2) show that the addition of GTP induce similar conformational transition in the two samples. The secondary structure content was calculated using the CD spectrum deconvolution software CDNN [51]. This software calculates the secondary structure by comparison with a CD database of known protein structures. The results of the spectra analysis are reported in table S1.

**
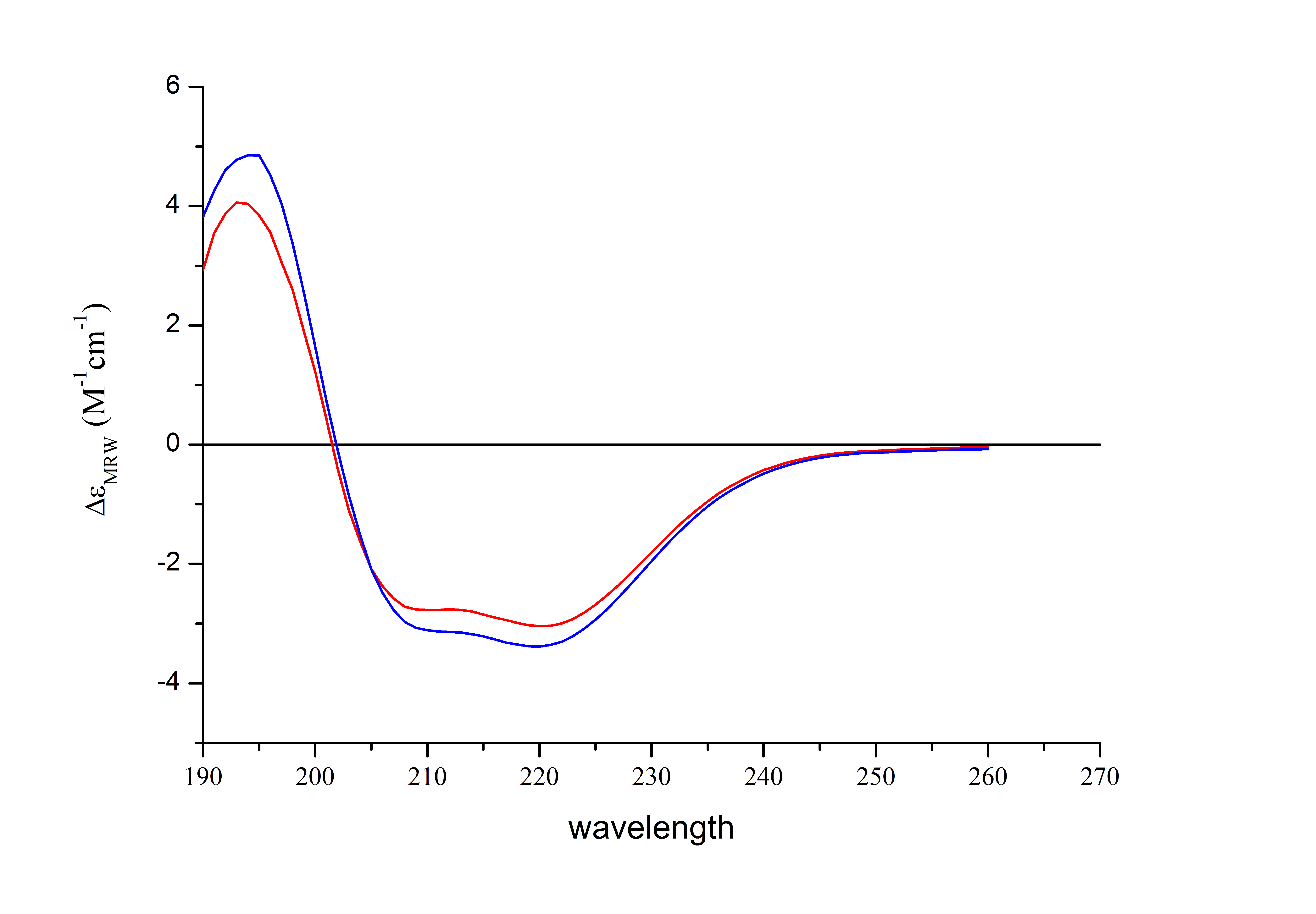
**

**Figure S2.** CD spectra of cysteine-less HydF mutant, before (blue) and after (red) GTP addition.

**Table S1.** **CD spectra analysis of HydF WT and cysteine-less mutant**

**Table:** percentages of secondary structure elements obtained from the fitting of the CD spectra by CDNN software. Uncertainty on the reported values are estimated to be of the order of few %.

|  | **WT** | | **cysteine-less** | |
| --- | --- | --- | --- | --- |
|  |  | +GTP |  | +GTP |
| helix | 32.9 | 30.0 | 33.0 | 30.4 |
| parallel | 8.7 | 11.8 | 7.8 | 9.8 |
| antiparallel | 8.7 | 9.1 | 8.9 | 9.6 |
| turn | 17.0 | 17.6 | 16.7 | 17.2 |
| random | 32.8 | 33.8 | 34.5 | 36.5 |
| Total | 100.1 | 102.3 | 100.9 | 103.5 |
|  | **Structural Changes**  **WT** | | **Structural Changes cysteine-less** | |
| Δhelix | -2.9 | | -2.6 | |
| Δparallel | +3.1 | | +2.0 | |
| Δantiparallel | +0.4 | | +0.7 | |
| Δturn | +0.6 | | +0.5 | |
| Δrandom | +1.0 | | +2.0 | |
| ΔTotal | +2.2 | | +2.6 | |

**Labeling yields of HydF mutants**

Labeling yield for the different mutants at the cysteine positions were evaluated by spin quantification of the EPR spectrum double integrals and comparison with those of standard solutions of the free spin labels, and are reported in table S1.

**Table S2.** Labeling yields of HydF mutants

| **Mutant** | **Yield (%) with MTSSL** | **Yield (%) with 5-MSL** |
| --- | --- | --- |
| S35C | 85 | 60 |
| S38C | 85 | 60 |
| T44C | 80 | 65 |
| V71C | 55 | 35 |
| R88C | 80 | 20 |
| A89C | 15 |  |
| T164C | 75 | 60 |
| I175C | 90 | 70 |
| V261C | 70 |  |
| D340C | 45 |  |
| L341C | 45 |  |
| C356 | 55 | 35 |

**Effects of K^+^ on the change of the CW-EPR spectrum of spin labelled R88C upon GTP addition**

1 mT

**Figure S3**: *Top:* CW-EPR spectra at room temperature of the mutant R88C in the absence (blue) and in the presence (red) of GTP, when potassium is not added to the buffer. *Bottom:* comparison between CW-EPR spectra of R88C in the absence of potassium (blue) and when 50 mM potassium is added to the buffer before (brown) or after (orange) the addition of GTP.

**Validation of the distance distributions**

**V261C**

**T164C-V261C**

**
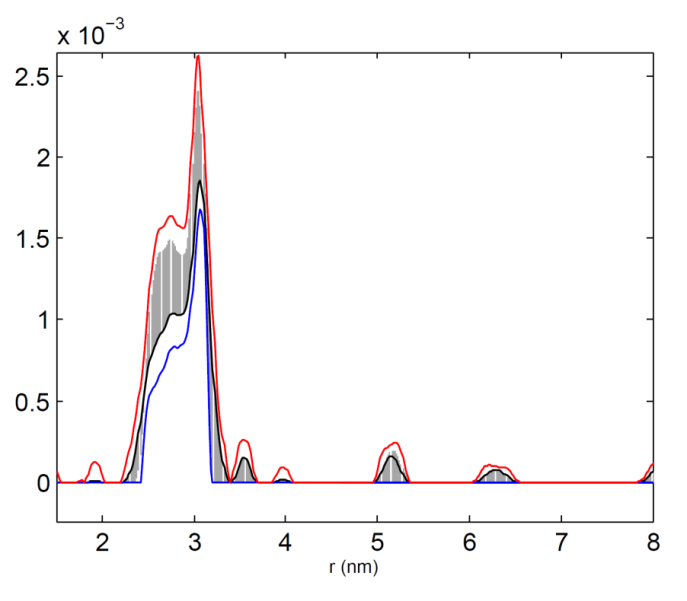

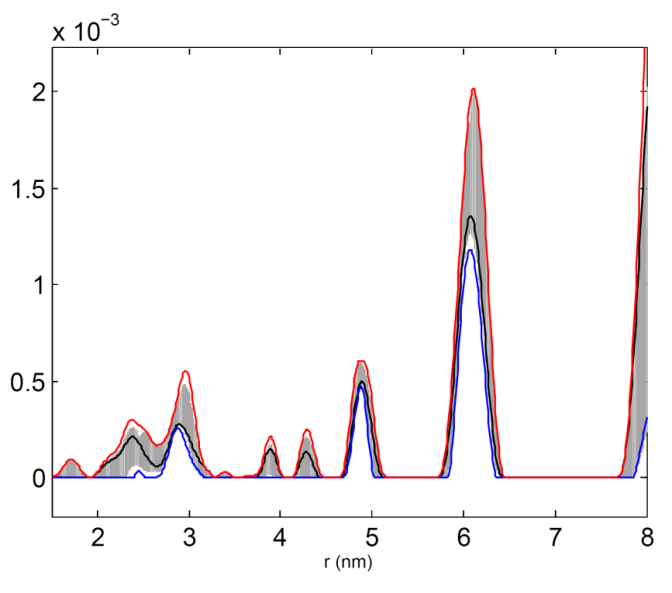
**

**
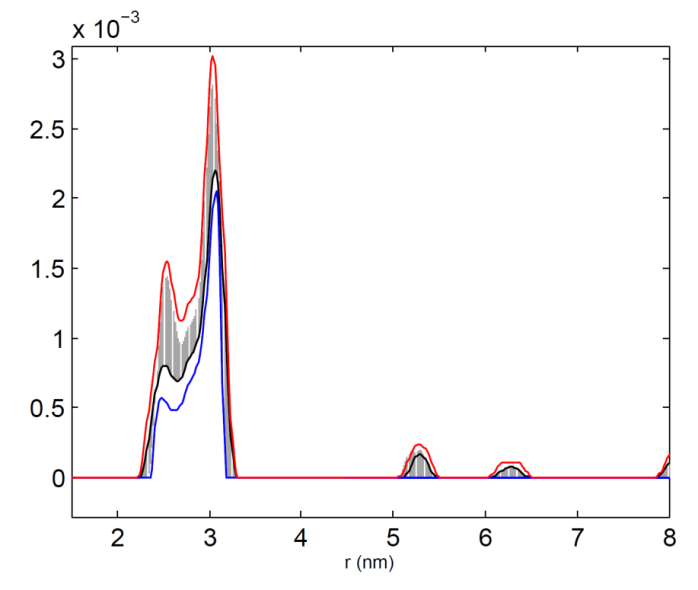

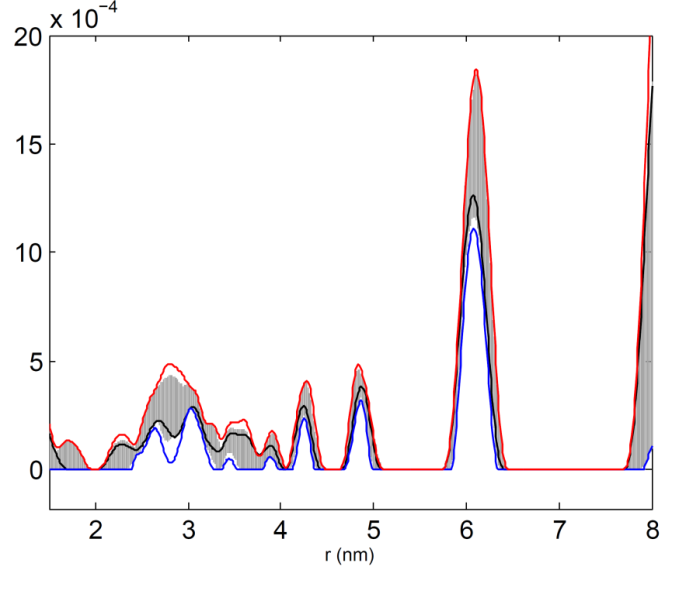
**

**Figure S4**: Validations of the distance distributions performed with DeerAnalysis2015 for the investigated mutants in the absence (*top*) and in the presence (*bottom*) of GTP. The black lines represent the distribution with the best r.m.s.d. after the validation procedure, while grey bars indicate the full variation of the probability for each distance over all trials. The other lines represent the mean value of the probability minus (blue) or plus (red) two times the standard deviation.

**Simulation of CW-EPR spectra for spin labeled R88C mutant**

The simulation of EPR spectra can provide information on the protein backbone dynamics at the different sites, and also a multicomponent analysis can determine the relative amounts of the different components in a spectrum. We performed an analysis of the EPR spectra of the MTSSL label at position 88 since it proved to be the most sensitive to GTP addition.

First we analysed the different components of the R88C-MTSSL spectrum to determine the extent of the changes induced by GTP. Since the experimental spectra clearly showed that only two components were present and that the addition of GTP likely shifted only the relative abundance, but not the lineshape, we were able to separate them by a properly weighed subtraction of the spectra with and without GTP: in Fig. S5, in the center we report the simulations of the individual components, the parameters of the simulation are reported in table S3. The two spectral shapes were simulated using a program based on the stochastic Liouville equation and adopting the MOMD model as standard for spin labeled proteins. The starting values for the principal components of the g and ^14^N hyperfine (A) tensors of the TOAC label were obtained from fitting of the frozen solution spectra. The values of the magnetic (g and A) and diffusion (D) tensors, their relative orientation (Ω_D_), and the order parameter (S), were then refined by simplex fitting. The two simulated lineshapes were then normalized to the same number of spins and used to obtain the simulation of the original experimental spectra as shown in Fig.S5 in the bottom part. The spectrum of the apo-protein is simulated by equal amounts of the two components (55% broad; 45% narrow), while the spectrum in the presence of GTP has a predominance of the narrow component (30% broad / 70% narrow). Given that the simulations are not perfect, an uncertainty of ±5% in the relative amounts of the two components can be estimated. Overall, the simulations suggest that the addition of GTP affects roughly a fourth of the protein at position 88.

As regards the analysis of the backbone dynamics, the diffusion tensors of the two components are both isotropic, the narrow component has a slightly faster diffusion (lower rotational correlation time) than the broad one, and the marked change in spectral width can be ascribed to the tumbling around different axes. The diffusion parameters suggest that no marked change in the backbone dynamics takes place upon GTP addition. We also estimated the overall rotational correlation time of the HydF dimer; the diffusion tensor is axial with slightly faster rotation around the “helical” axis of the dimer: D_||_ = 3.65 MHz and D_⊥_ = 2.05 MHz, this corresponds to a τ_HydF_ = 1/6(D_||_D_⊥_)^1/2^ = 61 ns. This value is higher than those obtained from the simulations, indicating that the overall tumbling of the protein should not strongly affect the lineshape of the EPR spectra.


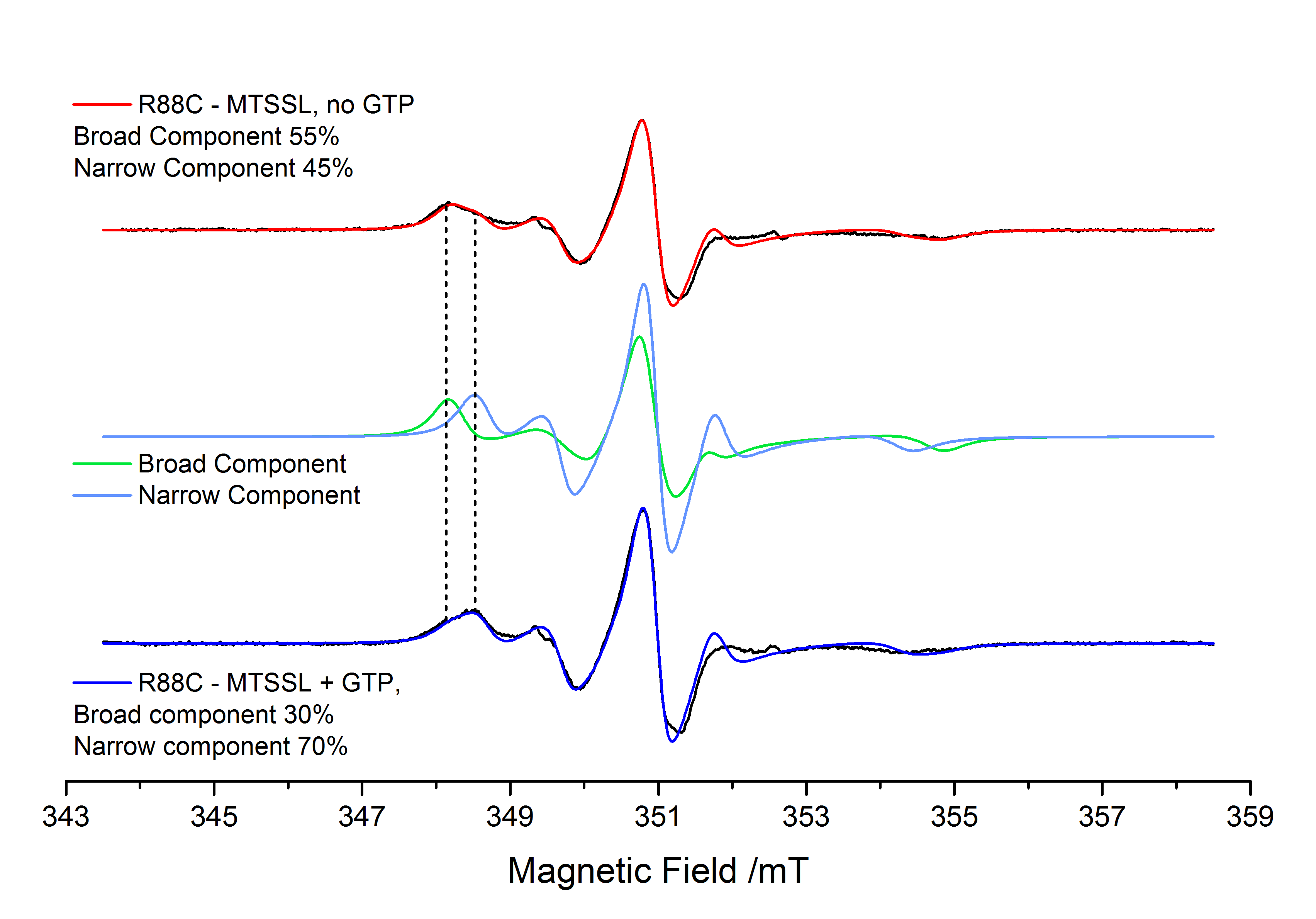


**Figure S5**. *Top*: spectrum *and simulation of R88C-MTSSL; bottom: spectrum and simulation of R88C-MTSSL with GTP; center*: simulate individual components. The dashed lines help highlight the main features of the two components.

**Table S3.** **Parameters obtained from the fitting of the individual components of R88C.**

|  | g_xx_ | g_yy_ | g_zz_ | A_xx_ | A_yy_ | A_zz_ | D | τ | Ω_D_ | S |
| --- | --- | --- | --- | --- | --- | --- | --- | --- | --- | --- |
| Broad | 2.0088 | 2.0070 | 2.0030 | 7.9 G | 5.4 G | 36.2 G | 11 MHz | 15 ns | 0°/0°/0° | 0.60 |
| Narrow | 2.0088 | 2.0070 | 2.0030 | 8.0 G | 5.4 G | 35.0 G | 31 MHz | 5 ns | 0°/37°/0° | 0.60 |

**ITC measurements**

ITC measurements of GTPγS binding to wild type HydF were carried out at 25 °C on a MicroCal OMEGA ultrasensitive titration calorimeter. The samples, in 25 mM Tris-HCl pH 8.0, 200 mM KCl, and 1 mM MgCl­_2_ buffer solution, were degassed before measurements. A 500 mM stock of GTPγS was titrated into a protein solution of 75 μM. Data were collected automatically and then analyzed with a Windows-based Origin software package supplied by MicroCal.

**Figure S6.** ITC measurements of GTPγS binding to wild type HydF 25 °C. Experimental data (circles) are interpolated by the curve (red) calculated with the parameters reported in the table S3 reported below.

**Table S4. GTPγS binding to wild typeHydF monitored by Isothermal Titration** Calorimetry (ITC)

| Sample | titrant | n | ΔH (cal mol-1) | ΔS (cal mol-1 deg-1) | K_D_ (μM) |
| --- | --- | --- | --- | --- | --- |
| HydF | GTPγS | 0,594 | -2873 ± 28,36 | 17,9 | 0,96 ± 0,09 |

**Table S5.** List of primers used in this study

| **Primer name** | **Primer sequence** |
| --- | --- |
| C91S_for | 5′-TCTACAGGGCAGATTCTGGAATTCTCGTGAC-3′ |
| C91S_rev | 5′-GTCACGAGAATTCCAGAATCTGCCCTGTAGA-3′ |
| C302S_for | 5’-GTCATCATGGAAGGCAGCACCCACAGACCTC-3’ |
| C302S_rev | 5’-GAGGTCTGTGGGTGCTGCCTTCCATGATGAC-3’ |
| C353S_for | 5’-CTTATCATCCACAGCGGTGGATGTATTCTG-3’ |
| C353S_rev | 5’-CAGAATACATCCACCGCTGTGGATGATAAG-3’ |
| C353S_C356S_for | 5’-CCACAGCGGTGGAAGTATTCTGAACCGTTC-3’ |
| C353S_C356S_rev  S35C_for  S35C_rev  S38C_for  S38C_rev | 5’-GAACGGTTCAGAATACTTCCACCGCTGTGG-3’  5'-GTTGGTCAGAACGTGTGCATCGTGAGCGATTAT-3'  5'-ATAATCGCTCACGATGCACACGTTCTGACCAAC-3'  5'-TGTCCATCGTGTGCGATTATGCGGGAA-3'  5'-TTCCCGCATAATCGCACACGATGGACA-3' |
| T44C_for | 5’-AGCGATTATGCGGGAACATGCACCGATCCCGTCTACAA-3’ |
| T44C_rev | 5'-TTGTAGACGGGATCGGTGCATGTTCCCGCATAATCGCT-3' |
| V71C_for | 5'-CCTGGACTCGACGACTGTGGAGAACTTGGAAGA-3' |
| V71C_rev | 5'-TCTTCCAAGTTCTCCACAGTCGTCGAGTCCAGG-3' |
| R88C-C91S_for | 5′-GGCAAGGTGCGTGTTCTACTGCGCAGATTGTGGAATTCTCG-3′ |
| R88C-C91S_rev | 5′-CGAGAATTCCACAATCTGCGCAGTAGAACACGCACCTTGCC-3′ |
| A89C-C91S_for | 5'-GGAGGGTGTTCTACAGGTGCGATTCTGGAATTCTC-3' |
| A89C-C91S_rev | 5'-GAGAATTCCAGAATCGCACCTGTAGAACACCCTCC-3' |
| T164C_for | 5’-GATTCGACGATATCGGGAAGTGCATCTCCGAAATTCTTCCGGG-3’ |
| T164C_rev | 5’-CCCGGAAGAATTTCGGAGATGCACTTCCCGATATCGTCGAATC-3’ |
| I175C_for | 5’-CCGGGTGATGAAGAGTGTCCTTACCTCGGTGATC-3’ |
| I175C_rev | 5’-GATCACCGAGGTAAGGACACTCTTCATCACCCGG-3’ |
| V261C_for | 5'-TGATGTCCCGGAAGACTGCGAACTCACCACCTTTT-3' |

| **Primer name** | **Primer sequence** |
| --- | --- |
| V261C_rev | 5'-AAAAGGTGGTGAGTTCGCAGTCTTCCGGGACATCA-3' |
| D340C_for | 5'-CCGGAAAAGATTTTCCTTGTCTTGAGGAAATAGAAAACGC-3' |
| D340C_rev | 5'-GCGTTTTCTATTTCCTCAAGACAAGGAAAATCTTTTCCGG-3' |
| L341C_for | 5'-CCGGAAAAGATTTTCCTGATTGTGAGGAAATAGAAAACGC-3' |
| L341C_rev | 5'-GCGTTTTCTATTTCCTCACAATCAGGAAAATCTTTTCCGG-3' |
